# Clinical value of neutrophil-to-lymphocyte ratio and prognostic nutritional index on prediction of occurrence and development of diabetic foot-induced sepsis

**DOI:** 10.3389/fpubh.2023.1181880

**Published:** 2023-10-25

**Authors:** Bing Sun, Yimin Chen, Yulin Man, Yu Fu, Jianchang Lin, Zhaohong Chen

**Affiliations:** ^1^Burn & Wound Repair Department, Fujian Medical University Union Hospital, Fuzhou, China; ^2^Fujian Burn Institute, Fujian Medical University Union Hospital, Fuzhou, China; ^3^Fujian Burn Medical Center, Fujian Medical University Union Hospital, Fuzhou, China; ^4^Fujian Provincial Key Laboratory of Burn and Trauma, Fujian Medical University Union Hospital, Fuzhou, China

**Keywords:** diabetic foot ulcers, NLR, PNI, sepsis, T2DM

## Abstract

**Background:**

Diabetic foot-induced sepsis is a serious complication associated with increased disability and mortality in hospitalized patients. Early prediction of admission and detection effectively improve treatment options and prevent further deterioration. This study aims to evaluate the clinical value of the neutrophil-to-lymphocyte ratio (NLR) and prognostic nutritional index (PNI) to predict the risk of sepsis in patients with diabetic foot ulcers (DFU).

**Methods:**

Retrospective analysis was performed on 216 patients who were admitted to the Fujian Medical University Union Hospital between January 2015 and December 2022. Patients with DFU were divided into the non-sepsis (*n* = 166) and the DFU-induced sepsis (*n* = 50) groups. The independent factors of DFU-induced sepsis were determined by univariate and multivariate logistic regression analyses. A receiver operating characteristic (ROC) curve was performed to compare the area under the curves (AUC) of PNI and NLR.

**Results:**

Multivariate logistic regression analysis revealed that the PNI, NLR, international normalized ratio (INR), thrombin time (PT), and C-reactive protein (CRP) were independent prognostic factors for DFU-induced sepsis. After adjusting for potential confounders, the adjusted odds ratios of NLR for DFU-induced sepsis were 1.121 (1.072–1.172), 1.132 (1.077–1.189), and 1.080 (1.022–1.142), while those of PNI were 0.912 (0.873–0.953), 0.902 (0.856–0.950), and 1.004 (1.001–1.006). Moreover, the AUC of NLR was significantly greater than that of CRP (0.790, 95% CI: 0.689–0.891, *p* < 0.001 vs. 0.780, 95% CI: 0.686–0.873, *p* < 0.001).

**Conclusion:**

NLR and PNI have been regarded as readily and independently predictive markers in patients with DFU-induced sepsis. NLR is critical for the early detection and effective treatment of DFU-induced sepsis and is superior to CRP.

## 1. Introduction

Type 2 diabetes mellitus (T2DM) is characterized by metabolic disorders and a systemic chronic inflammatory response induced by prolonged hyperglycemia (1), which can cause a variety of consequences such as peripheral neuropathy, peripheral vascular disease, and foot ulcers (2). Patients with T2DM are more susceptible to the majority of infectious diseases (3). According to studies, infections such as lung infection, sepsis, urinary tract infection, and cellulitis are believed to be more common in patients with T2DM than non-diabetics (4). Diabetic foot ulcers (DFU) are major problems that affect both individuals and healthcare systems (4, 5). In addition to T2DM, other rare etiologies of foot ulcers include Hereditary Sensory and Autonomic Neuropathy (6) and infrapopliteal arterial disease (7). DFU is one of the most serious complications of diabetes, contributing significantly to disability and death (8). It is reported that the lifetime risk for a diabetic patient to develop a foot ulcer is 15–25% (9). Once developed, diabetic foot ulcers confer a high risk of below-knee amputation. Recent decades have witnessed an 85% increase in diabetes-related lower extremity amputations (10, 11). Parallel to the increasing prevalence of multidrug-resistant bacteria, patients with DFU encounter a markedly elevated risk of general infection, resulting in considerable morbidity and mortality (12). Beyond diabetes management, patients are additionally encumbered by foot-related complications of the disease (13).

Sepsis is a potentially fatal illness or complication induced by a defective host immune response to infection, with significant morbidity and death rates worldwide (14). Sepsis remains one of the most difficult and expensive diseases to treat (15), despite the continuous refinement of treatment strategies and advancements in medical equipment. Sepsis and sepsis shock are serious complications that occur in patients with DFU who are more susceptible to infection (16), increasing the risk of non-traumatic amputation, multiple organ failure, and even death.

The prognostic nutritional index (PNI) is a comprehensive and novel biomarker of inflammation based on albumin (ALB) levels and lymphocytes (17). The neutrophil-to-lymphocyte ratio (NLR) and platelet-to-lymphocyte ratio (PLR) are simple and composite markers of inflammation and nutritional status with high stability and usability. Several studies have suggested that PNI is a reliable biomarker for predicting early prognosis in patients with malignant tumors (18, 19). In addition, previous studies have demonstrated that PNI or NLR is a biomarker for predicting survival and mortality rates of patients with sepsis-induced acute kidney injury (AKI) (20, 21). In addition, procalcitonin (PCT) and C-reactive protein (CRP) are known to be used to diagnose sepsis and predict its severity and mortality in patients (22, 23). Nevertheless, these indicators were easily disturbed by myocardial damage, coronary artery disease, autoimmunity, and tumors (24). Since sepsis is an acute complication that affects survival rate, no research has been performed to assess the predictive usefulness of PNI and NLR in patients with DFU. Therefore, the purpose of this research is to understand the significance of the predictive values of PNI and NLR in DFU-induced sepsis.

## 2. Materials and methods

### 2.1. Methods and materials

Data on 216 patients with DFU were collected retrospectively at the Fujian Medical University Union Hospital between January 2015 and December 2022. The inclusion criteria were as follows: (1) enrolled patients should be admitted without sepsis and (2) enrolled patients with DFU discharged from the hospital should have a DFU primary diagnosis code in the first or second diagnostic code. Furthermore, sepsis or sepsis shock should not be the first diagnosis code or be recorded before a DFU diagnosis code. The exclusion criteria were as follows: (1) patients younger than 18 years; (2) patients with missing clinical and laboratory data; (3) evidence of circulatory ulcers of the lower limbs caused by malnutrition, varicose veins, or tumors; and (4) evidence of foot ulcers caused by various diseases.

### 2.2. Clinical evaluation and definition

(1) Sequential organ failure assessment (SOFA) score ≥ 2 and probable infection are the criteria for sepsis according to the third international consensus (25).
(2) DFU (26): Diagnostic criteria for DFU are included in both the 2017 edition of the China Guide for Prevention and Control of Type 2 Diabetes and the China Guide for Prevention and Management of Diabetes (2019 edition).

### 2.3. Data collection and laboratory measurements

The electronic medical record system of Fujian Medical University Union Hospital was used to collect all patient data. The following information was extracted: (1) demographic parameters such as age, sex, and body mass index (BMI); (2) vital signs such as systolic blood pressure (SBP) and diastolic blood pressure (DBP); (3) complications such as hypertension, cardiovascular disease, peripheral vascular disease, chronic liver disease (CLD), and chronic kidney disease; and (4) laboratory results obtained within 24 h of admission, such as procalcitonin (PCT), C-reactive protein (CRP), platelet-to-lymphocyte ratio (PLR), neutrophil-to-lymphocyte ratio (NLR), prognostic nutrition index (PNI), white blood cell (WBC) count, hemoglobin A1c (HbA1c), ALB, total bilirubin (TBIL), direct bilirubin (DBIL), activated partial thromboplastin time (APTT), prothrombin time (PT), international normalized ratio (INR), serum sodium, serum calcium, serum creatinine, aspartate aminotransferase (AST), ALT, blood urea nitrogen (BUN), PCT, and CRP. PNI was calculated as serum ALB (g/L) + 5 lymphocyte count (10^9^/L) (27); and PLR and NLR were calculated by dividing absolute platelet count by absolute lymphocyte count and neutrophil count by absolute lymphocyte count, respectively (28).

### 2.4. Data analysis

Baseline characteristics and laboratory data of all patients were stratified based on whether they had sepsis or not. The research variables were divided into continuous and classified variables, and the Shapiro–Wilk test was used to determine whether the variables conformed to the normal distribution. Mean and standard deviation were used to express continuous variables with a normal distribution, while median and interquartile ranges were used to depict skewed distributions. The independent-sample test and the Wilcoxon Mann–Whitney test were used in univariate analysis to compare the continuous variables between the non-sepsis and the DFU-induced sepsis groups. The chi-square test or Fisher exact test was used to compare the frequency of classified variables as a percentage. The variables associated with DFU-induced sepsis were identified using multivariate logistic regression analysis, and the confounding factors were adjusted to determine the predictive value of the PNI and NLR on the occurrence of DFU-induced sepsis. The accuracy of various DFU-induced sepsis indicators was further investigated using receiver operating characteristic (ROC) curve analysis. To compare the area under the ROC curve (AUC), the *z*-test was used. SPSS 25.0 and R version 4.2.1 were used for all analyses, and the value of *p* of 0.05 was considered statistically significant.

## 3. Results

### 3.1. Demographic profiles of patients with DFU

**Figure 1:**
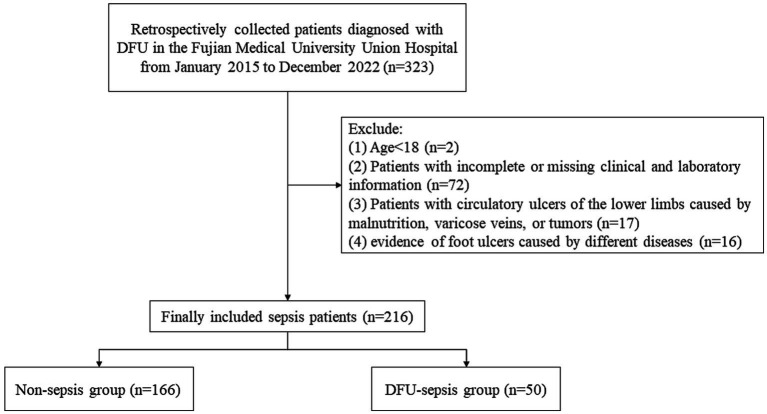
Flowchart of patient selection.

Figure 1 illustrates the flow chart of patient registration. A total of 216 patients with DFU were categorized into two groups based on the research objective (whether sepsis eventually occurred). Among them, 50 patients were diagnosed with sepsis/septic shock. The patients’ ages ranged from 54 to 72 years, with an average age of 66 years. Women accounted for 14.8% of the total sample, while men comprised 85.2%. Table 1 presents the demographic and clinical characteristics of patients with DFU in both the non-sepsis and DFU-induced sepsis groups. There were no statistically significant differences in age, sex, BMI, SBP, DBP, qSOFA, or comorbidities such as cardiovascular disease, hypertension, diabetic peripheral neuropathy (DPN), diabetic retinopathy (DR), or chronic liver disease (CLD) (*p* > 0.05).

**Table 1:** Demographic characteristics between the non-sepsis group and sepsis group.

Variables	Total (*n* = 216)	Non-sepsis group (*n* = 166)	Sepsis group (*n* = 50)	Value of *p*
Sex, *n* (%)				0.299
Male	65 (30.1)	47 (21.8)	18 (8.3)	
Female	151 (69.9)	119 (55.1)	32 (14.8)	
Age (years)	66 (56, 73)	66.5 (57.5, 72.3)	66 (53.5, 71.5)	0.488
BMI (kg/m^2^)	23.41 (20.96, 25.63)	23.83 (21.52, 26.56)	22.96 (20.24, 25.61)	0.069
SBP (mmHg)	136 (125, 148)	135 (122, 150)	132 (125, 140)	0.963
DBP (mmHg)	78 (72, 82)	78 (72, 84)	76 (68, 82)	0.138
qSOFA	0 (0, 1)	0 (0, 1)	0 (0, 1)	0.638
Comorbidities, *n* (%)				
Cardiovascular disease	37 (17.1)	28 (13.0)	9 (4.2)	0.852
Hypertension	80 (37)	62 (28.7)	18 (8.3)	0.862
DPN	91 (42.1)	67 (31.0)	24 (11.1)	0.338
DR	23 (10.6)	17 (7.9)	6 (2.8)	0.724
CKD	17 (7.9)	8 (3.7)	9 (4.2)	<0.001
CLD	12 (5.6)	7 (3.2)	5 (2.3)	0.118

BMI, body mass index; SBP, systolic blood pressure; DBP, diastolic blood pressure; DPN, diabetic peripheral neuropathy; DR, diabetic retinopathy; CKD, chronic kidney disease; CLD, chronic liver disease.

### 3.2. Univariate and multivariate logistic regression analyses of clinical and laboratory results

Laboratory markers were employed to evaluate the risk prediction for both patient groups. According to our findings, patients in the sepsis group had weaker coagulation and inflammatory responses than those in the non-sepsis group. Table 2 summarizes the univariate clinical and laboratory data within 24 h of admission. There were significant differences in WBC count, hemoglobin, INR, APTT, PT, DBIL, serum calcium, ALB, PCT, CRP, PLR, NLR, and PNI between the non-sepsis and sepsis groups. Multivariate binary logistic regression analysis was used to identify potential predictors of sepsis in patients with DFU. As presented in Table 3, CRP, INR, PT, NLR, and PNI were all independent predictors of sepsis in patients with DFU. After controlling for potential confounding factors, we developed several models to evaluate the independent effects of NLR and PNI in patients with DFU-induced sepsis. The adjusted odds ratios of NLR for DFU-induced sepsis were 1.121 (95% CI: 1.072–1.172), 1.132 (95% CI: 1.077–1.189), and 1.080 (95% CI: 1.022–1.142), respectively, while those of PNI were 0.912 (95% CI: 0.873–0.953), 0.902 (95% CI: 0.856–0.950), and 1.004 (95% CI: 1.001–1.006) (Table 4).

**Table 2:** Laboratory characteristics of patients with DFU between the non-sepsis group and sepsis group.

Variables	Total (*n* = 216)	Non-sepsis group (*n* = 166)	Sepsis group (*n* = 50)	Value of *p*
WBC (10^9^/L)	9.08 (6.78, 13.76)	7.95 (6.56, 9.93)	14.91 (8.72, 19.84)	<0.001
Hemoglobin (g/dL)	115 (97.25, 131.75)	113 (102, 136)	117 (98, 124)	0.029
HbA1C (%)	9.80 (8.10, 11.70)	9.65 (8.12, 11.40)	9.80 (7.63, 11.57)	0.512
GLU (mmol/L)	8.52 (6.38, 12.92)	8.24 (5.54, 12.97)	8.50 (7.72, 11.56)	0.779
BUN (mmol/L)	5.6 (4.4, 7.8)	5.3 (4.3, 8.5)	6.3 (5.1, 7.7)	0.101
CRE (μmol/L)	71.0 (59.3, 92.5)	74.5 (61.2, 94.3)	83.0 (62.8, 102.5)	0.345
INR	1.02 (0.92, 1.11)	0.98 (0.92, 1.09)	1.06 (0.98, 1.15)	<0.001
APTT (s)	38.2 (34.7, 42.9)	35.9 (32.5, 41.0)	40.0 (37.5, 44.8)	<0.001
PT (s)	13.3 (12.4, 14.3)	12.8 (12.1, 14.1)	13.8 (12.9, 14.7)	<0.001
TBIL (μmol/L)	8.75 (6.03, 13.08)	8.00 (5.63, 10.83)	10.60 (5.93, 13.55)	0.102
DBIL (μmol/L)	2.7 (1.9, 4.4)	2.3 (1.7, 4.0)	3.5 (1.8, 4.0)	0.007
AST (U/L)	19 (14, 24)	20 (15, 28)	17 (13, 24)	0.214
ALT (U/L)	17.5 (12.0, 25.8)	22.0 (13.8, 30.0)	13.5 (10.3, 19.8)	0.312
Sodium (mg/L)	138.4 (135.5, 140.7)	139.2 (136.5, 141.4)	137.4 (134.0, 140.5)	0.082
Calcium (mg/L)	2.19 (2.08, 2.31)	2.24 (2.14, 2.30)	2.12 (1.93, 2.33)	<0.001
Albumin (g/L)	31.9 (27.1, 36.5)	32.8 (27.5, 37.4)	30.2 (26.8, 33.4)	0.001
PCT (ng/ml)	0.14 (0.05, 0.84)	0.11 (0.05, 0.48)	0.27 (0.09, 1.75)	<0.001
CRP (mg/L)	68.2 (10.8, 146.5)	32.5 (6.7, 93.3)	156.6 (103.0, 211.7)	<0.001
PLR	172.75 (115.70, 270.24)	152.67 (107.15, 237.63)	276.84 (137.29, 364.00)	<0.001
NLR	3.91 (2.21, 9.00)	3.42 (2.08, 6.27)	10.00 (4.96, 25.84)	<0.001
PNI	40.13 (32.98, 46.10)	41.78 (34.20, 47.16)	35.28 (29.86, 44.75)	<0.001

WBC, white blood cell; HbA1c, hemoglobin A1c; BUN, blood urea nitrogen; CRE, creatinine; APTT, activated partial thromboplastin time; PT, thrombin time; INR, international normalized ratio; AST, aspartate aminotransferase; ALT, alanine aminotransferase; PCT, procalcitonin; CRP, C-reactive protein; PLR, platelet-to-lymphocyte ratio; NLR, neutrophil-to-lymphocyte ratio; PNI, prognostic nutrition index.

**Table 3:** Multivariate logistic regression analysis of risk factors for sepsis in DF patients.

Variables	OR	95%CI	value of p
CRP	1.02	1.00–1.027	0.014
INR	0.001	0.001–3.839	0.016
PT	10.45	1.520–71.803	0.021
NLR	1.53	1.040–2.248	0.032
PNI	1.67	1.040–2.655	0.032

PT, thrombin time; INR, international normalized ratio; CRP, C-reactive protein; NLR, neutrophil-to-lymphocyte ratio; PNI, prognostic nutrition index.

**Table 4:** Association of PNI and NLR with sepsis in DFU patients.

Exposure	Non-adjusted OR	*p*	Adjusted OR	*p*
Model 1				
NLR	1.107 (1.063–1.154)	<0.001	1.121 (1.072–1.172)	<0.001
PNI	0.918 (0.880–0.956)	<0.001	0.912 (0.873–0.953)	<0.001
Model 2				
NLR	1.107 (1.063–1.154)	<0.001	1.132 (1.077–1.189)	<0.001
PNI	0.918 (0.880–0.956)	<0.001	0.902 (0.856–0.950)	<0.001
Model 3				
NLR	1.107 (1.063–1.154)	<0.001	1.080 (1.022–1.142)	0.007
PNI	0.918 (0.880–0.956)	<0.001	1.004 (1.001–1.006)	0.014

Model 1: adjusted for age and sex. Model 2: model 1 and diabetes, hypertension, cardiovascular disease, diabetic peripheral neuropathy, diabetic retinopathy, chronic liver disease, chronic kidney disease, BMI, SBP, DBP. Model 3: model 2 and WBC, Hemoglobin, HbA1C, GLU, BUN, CRE AST, ALT, INR, APTT, PT, TBIL, DBIL, sodium, calcium, ALB, and PCT.

### 3.3. ROC curve analysis of NLR and PNI

**Figure 2:**
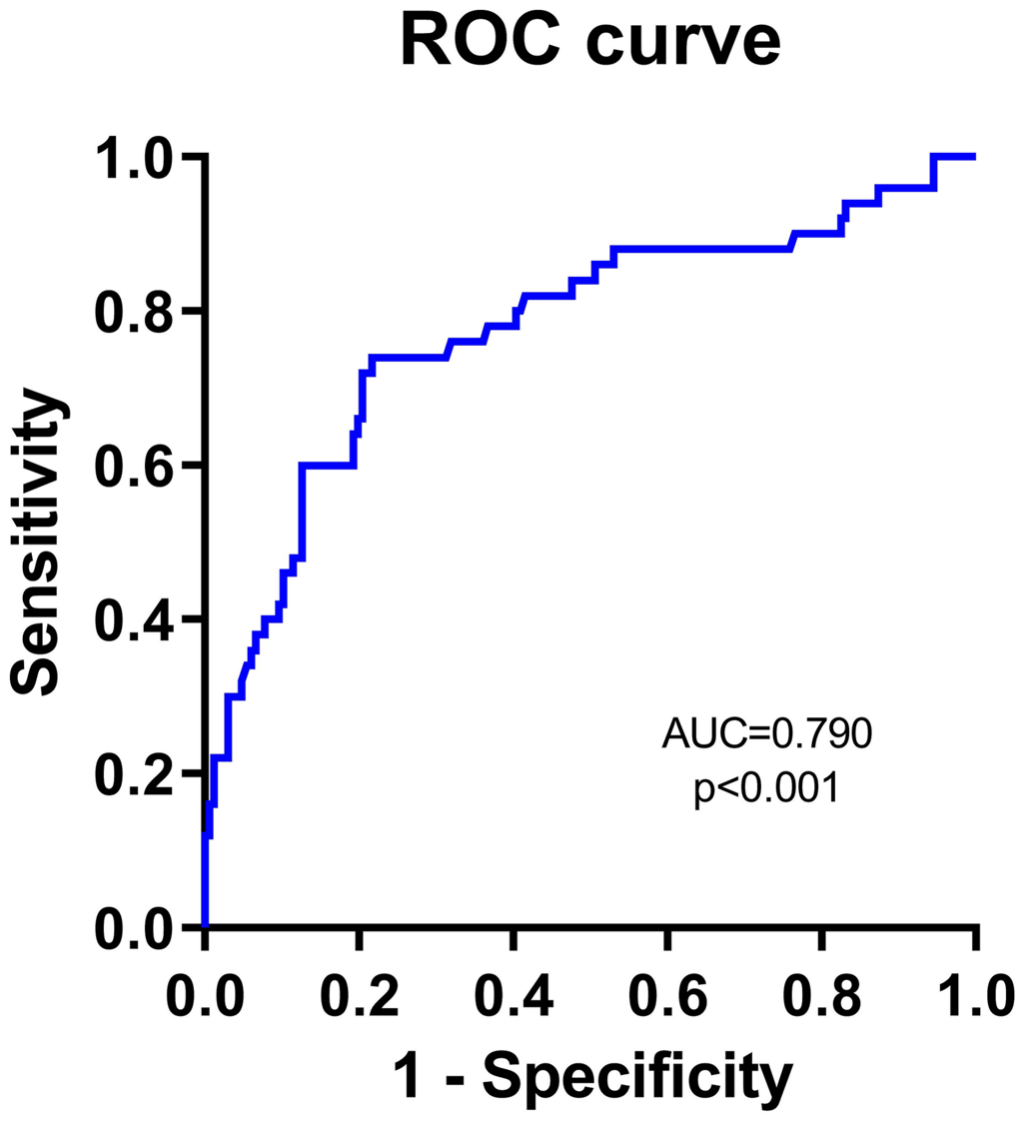
Receiver operating characteristic curve analyses of independent predictors for DFU-induced sepsis. The ROC curve of NLR in predicting DFU patients with sepsis.

**Figure 3:**
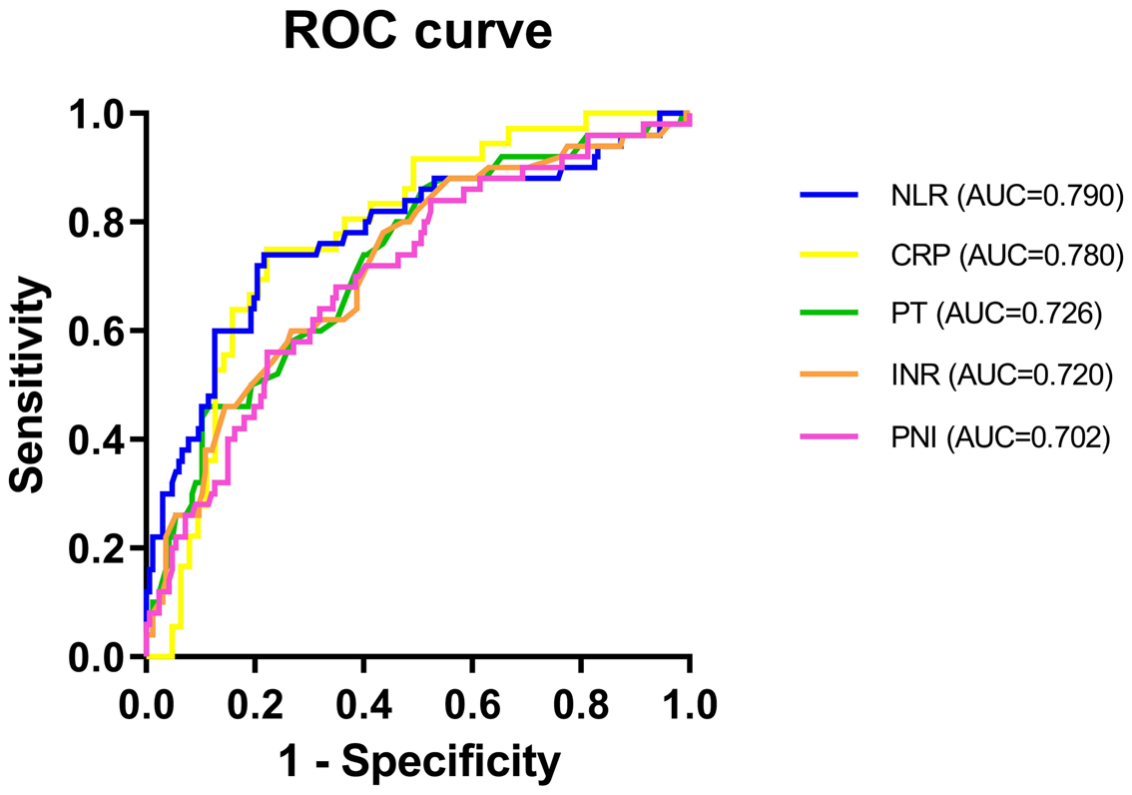
ROC curve of NLR, CRP, INR, PT, and PNI in predicting patients with DFU-induced sepsis. PT, thrombin time; INR, international normalized ratio; CRP, C-reactive protein; NLR, neutrophil-to-lymphocyte ratio; PNI, prognostic nutrition index.

To evaluate the predictive value of NLR and PNI for the incidence of DFU-induced sepsis, ROC curve analysis was performed, as shown in Figures 2, 3. Table 5 displays the results of the AUC with a 95% confidence interval in ROC analysis. The AUC of the NLR was 0.790 (95% CI: 0.689–0.891, *p* < 0.001), which was significantly greater than that of the CRP (0.780, 95% CI: 0.686–0.873, *p* < 0.001). Additionally, the AUC of PNI was a substantial predictor for DFU-induced sepsis (0.702, 95%CI: 0.619–0.785, *p* < 0.001).

**Table 5:** Receiver operating curve (ROC) for prediction in patients with sepsis.

Indicator	AUC	95% CI	*p*	Optimal cutoff value	Sensitivity (%)	Specificity (%)
CRP	0.780	0.686–0.873	<0.001	94.51	0.75	0.778
INR	0.720	0.590–0.807	<0.001	1.015	0.778	0.556
PT	0.726	0.601–0.813	<0.001	12.95	0.833	0.508
NLR	0.790	0.689–0.891	<0.001	6.814	0.75	0.825
PNI	0.702	0.619–0.785	<0.001	34.75	0.56	0.777

PT, thrombin time; INR, international normalized ratio; CRP, C-reactive protein; NLR, neutrophil-to-lymphocyte ratio; PNI, prognostic nutrition index.

## 4. Discussion

This study is the first study to demonstrate that sepsis in patients with DFU can be predicted by specific inflammatory markers. Our research yielded several important findings, such as NLR, PLR, and PNI were risk factors for sepsis in both patients without sepsis and those with DFU-induced sepsis; however, only NLR and PNI were independent risk factors for higher morbidity in DFU-induced sepsis. In addition, it was observed that higher NLR and lower PNI at admission are associated with DFU-sepsis morbidity.

Sepsis is the leading cause of death worldwide, with a mortality rate of more than 10%. It is caused by the host’s maladaptive response to infection (29). The diabetes-related complication is a major cause of hospitalization, disability, and death. Several studies have reported that patients with T2DM have a 2–6 times higher risk of sepsis (30) and a higher risk of sepsis-related morbidity and mortality (31) when compared with age-matched patients with non-T2DM. Furthermore, drug-resistant pathogen colonization may occur more frequently in patients with T2DM (32). The pathophysiological mechanism of DFU is still debated. Previous studies have identified diabetic foot as one of the serious complications caused by pathophysiological changes such as inflammation, endothelial dysfunction, and blood coagulation imbalance (33). The presence of immune cells in an environment of chronic hyperglycemia and hypertriglyceridemia caused chronic immune system disorders and impaired responses to acute infections and sepsis in patients with DFU. In addition, the coexistence of DFU and severe sepsis compromised RBC deformability, worsened microcirculation, accelerated the progression of organ dysfunction, and even worsened the long-term prognosis of patients with DFU with general infection and sepsis (34, 35). Sepsis has been reported to be an independent risk factor for lower limb amputations in patients with DFU (36), resulting in increased hospital readmissions, amputation rates, morbidity, and mortality (37). Therefore, early detection of high-risk patients with DFU-induced sepsis is critical for prediction and prevention.

PNI, which has a significant impact on the patient’s nutritional and immune status, was estimated using ALB levels and the total number of peripheral blood lymphocytes (18). Nutritional status is commonly used to indicate health status and predict infection. In patients with cancer, PNI is a reliable prognostic factor. Furthermore, PNI has emerged as a prognostic biomarker for patients with acute ischemic stroke (38), sepsis-induced AKI (20), and coronary heart disease (39). Li et al. reported that the presence and severity of neonatal septicemia were negatively and independently correlated with PNI (40). Patients with DFU-induced sepsis had significantly lower PNI than those without sepsis (41.78, 34.20–47.16 vs. 35.28, 29.86–44.75, respectively, *p* < 0.001).

Immunologic function, inflammation, and nutritional status play crucial roles in the pathogenesis of sepsis. NLR is a well-established biomarker of systemic inflammation that is essential for sepsis diagnosis and prognosis. Due to the following primary reasons, an aberrant inflammatory response resulted in severe infections and sepsis: 1. Neutrophils secrete pro-inflammatory mediators, which induce endothelium and organ damage, as well as an increase in the risk of severe consequences and limb amputation (41); 2. Increased platelet levels, one of the inflammatory mediators, may indicate prothrombotic activity and chronic inflammatory conditions by stimulating megakaryocytes (42, 43); and 3. Lymphocytes can exert anti-inflammatory effects by secreting anti-inflammatory substances such as interleukin-10. As such, when sustaining oxidative DNA damage in hyperglycemic conditions, lymphocytes can predispose the body toward an immunosuppressive state (44). The systemic inflammatory response causes changes in neutrophil, monocyte, and platelet counts. Numerous studies have suggested that NLR and PLR may predict systemic inflammation (45) and that these markers are highly sensitive to numerous diseases (46) and readily accessible in clinical practice (47, 48). Furthermore, NLR has a significant role in predicting COVID-19 pneumonia in patients with T2DM (49). Despite the close association between inflammation and DFU, no previous studies have focused on the role of NLR and PLR in assessing and predicting the presence of DFU-induced sepsis.

Numerous studies have shown that malnutrition, immunology, and inflammation all play important roles in the initiation and progression of diabetes (8), especially with regard to its long-term consequences. Higher levels of inflammatory mediators and catabolic hormones stimulate catabolism while weakening anabolism in DFU patients, resulting in severe malnutrition, immunosuppression, and worsened inflammatory responses. In this study, the immune-nutritional marker PNI (AUC = 0.702) and the inflammatory marker NLR (AUC = 0.790) have been shown to increase the accuracy of predicting the development of sepsis. It should be noted that in our study, PCT could not be utilized as an independent predictor of sepsis in DFU patients, and there was no significant difference in multivariate logistic regression analysis. This phenomenon could be explained by PCT being less sensitive to early local infection, mild infection, and chronic inflammatory responses but more sensitive to acute systemic inflammatory response syndrome. According to studies, PCT is an independent prognostic factor for all-cause mortality in patients with sepsis (50). Furthermore, PCT can be used to diagnose bacterial sepsis or septic shock (51) and is a valuable marker for the diagnosis of patients with T2DM who have DFU infection (52). Therefore, more samples need to be collected to confirm the early predictive effect of PCT.

Our study has a few limitations, which are as follows: (1) This is a single-center, retrospective study with a small sample size. Therefore, more prospective studies are required to validate our findings and confirm the predictive effectiveness of NLR and PNI in DFU-induced sepsis. (2) While investigating the risk factors for sepsis in patients with DFU, not all of them were considered. (3) Only the initial serological indexes within 24 h of admission were included; however, changes in the index of patients with DFU during hospitalization could not be dynamically analyzed. (4) Owing to the lack of corresponding follow-up data, we were unable to analyze the contribution of risk factors to the survival status on follow-up of patients.

## 5. Conclusion

In clinics, assessing the risk of developing sepsis in DFU and conducting a hospital-based study have remained an ongoing challenge. In our study, CRP, INR, PT, NLR, and PNI were observed to be independent predictors of sepsis in patients with DFU. Furthermore, the inflammatory marker NLR has a higher diagnostic value than the conventional marker CRP, indicating that they may have complementary benefits and improve the accuracy of early DFU-induced sepsis prediction.

## Data availability statement

The raw data supporting the conclusions of this article will be made available by the authors, without undue reservation.

## Ethics statement

The studies involving humans were approved by the Ethics Committee of the Fujian Medical University Union Hospital (No. 2022KY222). The studies were conducted in accordance with the local legislation and institutional requirements. Written informed consent to participate in this study was not required from the participants in accordance with the national legislation and the institutional requirements. No potentially identifiable human images or data are presented in the manuscript.

## Author contributions

BS conceived of the study and drafted the manuscript. YC, YM, and YF gathered and processed the data. JL, ZC conception, supervision, and revised the manuscript. All authors contributed to the article and approved the submitted version.
